# Education Research: The Landscape of Adult Neurology Residency Didactic Curricula in the United States: A Cross-Sectional Survey

**DOI:** 10.1212/NE9.0000000000200205

**Published:** 2025-03-07

**Authors:** Harry W. Sutherland, Christine E. Gummerson, John Encandela, Fábio A. Nascimento, Jeremy J. Moeller

**Affiliations:** 1 Department of Neurology, Yale School of Medicine, New Haven, CT;; 2 Department of Psychiatry, Yale School of Medicine, New Haven, CT;; 3 Center for Medical Education, Yale School of Medicine, New Haven, CT; and; 4 Department of Neurology, Washington University School of Medicine, St. Louis, MO.

## Abstract

**Background and Objectives:**

Neurology residents learn through work-based learning, self-directed learning, and formal didactics. US residency program didactic curricula comply with national standards, but there may be wide design variation. No organization collects data on curricula, so the landscape of educational practices is unclear. This makes it difficult for program directors (PDs) to compare their approach with their peers' and identify methods of interest used elsewhere. We sought to describe existing curricular designs, examine features associated with resident attendance, and evaluate utilization of asynchronous learning.

**Methods:**

A survey was designed and validated following established standards. Anonymous online surveys were distributed by email to PDs of US adult neurology programs in April 2023, with responses collected until July 2023. Responding program characteristics were compared with national norms to check representativeness. Descriptive statistics were used to delineate the range of curricular designs. Associations between didactic choices, program characteristics, and attendance were analyzed using nonparametric methods.

**Results:**

Seventy-six (42.0%) of 181 programs responded. Respondents were more likely to be academically based (82% vs 63% nationally, *p* = 0.004) and with larger mean class sizes (7.9 vs 6.1, *p* < 0.001). Daily/noon conference (NC) models were more common than academic half-day (AHD) (63% vs 37%), and lectures predominated. AHD was less common in academic centers (30.0% vs 71.4% elsewhere; *p* = 0.004), the Northeast (14% vs 52.2% elsewhere; *p* = 0.001), and larger classes (6.4 vs 8.8 NC; *p* = 0.022). 75% reported that residents were at least “somewhat” responsible for pagers during conference—with various providers providing coverage. The reported attendance was 63.6 ± 22.0% (range 10%–90%). Attendance was not improved by food (*p* = 0.415) or AHD (*p* = 0.230), but it was improved by protected time (78% vs 58% unprotected; *p* < 0.001), fewer didactic hours (*p* = 0.031), and more PD-led sessions (*p* = 0.010). 75% of programs encouraged external asynchronous resource usage, and 65% developed internal materials—largely focused on examination preparation and neurophysiology.

**Discussion:**

The results of this survey describe the landscape of formal didactic curricula. Residency programs use a range of strategies to achieve their educational aims, although some elements are more common to certain program types and some were more successful at increasing resident attendance. Further study is needed to determine best practices from available methodologies.

## Introduction

As adult learners, neurology residents often learn by doing, with much of their knowledge deriving from their clinical work. There are several models for this work-based learning, including the experiential learning cycle^1^ and situated learning through “legitimate peripheral participation.”^2^ To augment and enhance clinical learning experiences, residency programs also incorporate structured didactic curricula into the resident experience for more formal, standardized graduate medical education (GME).

The Accreditation Council for Graduate Medical Education (ACGME) sets minimum requirements for GME curricula, but programs rightfully have a high degree of autonomy when designing and implementing formal instruction for their trainees in their specific educational context. Therefore, the structure and application of formal didactic instruction can vary from program to program. There is a wide range of available pedagogies, but options may include lectures, conferences, case-based discussions, simulation sessions, or laboratories.^3^

Residency program directors (PDs) have a community of peers to turn to when creating or adapting their educational programming, but there is currently no formal repository for didactic curricular design housed by the ACGME, Association of American Medical Colleges, or American Academy of Neurology. Consequently, it may be difficult for program leadership to (1) compare their own programmatic practices with national trends and (2) identify curricular practices developed elsewhere that may fit their individual program needs.

There have been a number of educational innovations published recently within the field of neurology—with a number of studies focusing on simulation sessions and the development of various asynchronous learning resources to facilitate self-directed learning.^4^ Despite numerous publications supporting individual tools, residency program attitudes toward asynchronous learning generally remain unclear, as do preferences for any specific instructional materials.

This observational, descriptive study aims to better define the landscape of adult neurology residency didactic curricula practiced in the United States through direct survey of residency program directors.

## Methods

### Survey Design

Authors H.W. Sutherland and C. Gummerson (neurology trainees) primarily generated the questionnaire items following published guidance on survey design.^5^ Questions were derived from these authors' individual experiences as residents and from familiarity with the experience of colleagues at other institutions, but formal focus groups were not conducted. Authors F.A. Nascimento and J. Encandela, who have extensive previous experience with survey design, provided feedback on these items and content validation. Author J.J. Moeller, a residency program director, piloted the survey. During this pilot testing, a think-aloud cognitive interview method was used to give author HS insights into response process for further item development before distribution.

### Survey Content

The first section of the survey concerned general characteristics of each program. Respondents were asked to provide background information about program affiliation, location, setting, and size. Subsequent items covered the specifics of didactic scheduling and instructional design, resident “protected time” during didactics, and the availability of food during sessions. Respondents were also asked to estimate average overall resident didactic attendance. The next 2 sections concerned practices around morning reports (MRs) and professor's rounds (PRs) specifically. Finally, program directors were asked to indicate attitudes and practices regarding internal and external asynchronous learning materials—including a list of preferred resources and the subject matter of any internally developed asynchronous learning tools.

A full PDF version of the survey is available for detailed review as a supplement to this article (eAppendix 1). Survey respondents were never asked to identify their residency program to mitigate privacy concerns, and individual responses were not tracked. All items were optional, allowing respondents to proceed past questions they had difficulty with or were not comfortable answering.

For participant characteristics, the authors defined US regions post hoc as “Northeast,” “Southeast,” “Midwest,” “Southwest,” and “West,” similar to the categorization scheme used by the AMA. A state-by-state breakdown is provided as a supplement to this article (eAppendix 2). Military program affiliations were arbitrarily coded as “hybrid” in addition to programs classified as “community-based university affiliated” on the American Medical Association (AMA) FREIDA database. This database already provides labels for academic and community programs. Using US Census Bureau data, residency programs located in places with a population >100,000 were defined as “urban” while those located in places with a population <10,000 were defined as “rural” and the remainder were classified as “suburban.”

### Survey Distribution

A complete list of adult neurology residency training programs based in the United States was obtained from the AMA FREIDA database. Program director and coordinator names plus email addresses were obtained from this database, along with academic affiliation, geographic location, and number of resident positions per class. Where these data were incomplete, individual program websites were queried and additional contact information was obtained from Doximity.com as needed.

The survey was published using Qualtrics XM, a secure, web-based questionnaire platform. The survey was distributed to adult neurology residency program directors individually by email using an anonymous, nontrackable link. Residency program coordinators were copied on these emails. There was no incentive provided to participate in this study. Email invitations to complete the survey were sent between mid and late April 2023. Follow-up reminders were sent in mid to late May 2023 and again in early July 2023. After some responses had been collected, the authors were made aware that answer choices using the slider bar were not displaying correctly for 2 items, making the options to select an odd number of residents per class and to select “4” professor's rounds sessions per month unavailable. This was corrected during the time the survey was active—after 33 of 76 responses were collected. After conclusion of the survey, we reviewed the data to ensure that this change did not have a meaningful effect on any of the reported results.

### Statistical Methods

Simple descriptive statistics were used for Tables 1–5. All statistical comparisons performed in this analysis were nonparametric to account for any non-normality or inequality of variances in the data. The χ^2^ test for goodness of fit was performed to compare the distribution of key nominal program characteristics in Table 1. In this same table, class size distributions were compared using a one-sample Kolmogorov-Smirnov (K-S) test. Throughout the text, numerical outcomes between 2 groups were tested using the Mann-Whitney *U* test while the Kruskal-Wallis *H* test was used among 3 or more groups. Specifically for Table 3, Mann-Whitney *U* tests were performed to compare the distribution of daily/noon conference model utilization in a pairwise manner between dichotomized groups for each stratum analyzed per characteristic feature of interest (e.g., academic vs else, community vs else, and hybrid vs else in the “affiliation” group). Comparisons between 2 continuous variables were made using Spearman rank correlation. Comparisons between categorical groups and categorical outcomes were made using Fisher exact tests. Bonferroni corrections were performed to account for multiple comparisons. All statistical tests were executed using IBM SPSS Statistics software version 28.0.1.1 (14).

**Table 1:** Residency Program Characteristics

Characteristic	Respondents (n = 76)	All US adult neurology programs (n = 181)	*p *Value^a^
Institutional affiliation, n (%)			0.004*
Academic	62 (82)	113 (63)	
Community	4 (5)	17 (9)	
Hybrid	10 (13)	51 (28)	
Region, n (%)			0.147
Northeast	28 (37)	46 (25)	
Southeast	21 (28)	50 (28)	
Midwest	13 (17)	40 (22)	
West	8 (11)	21 (12)	
Southwest	6 (8)	24 (13)	
Population setting, n (%)^b^			0.003*
Urban	56 (74)	136 (75)	
Suburban	14 (18)	41 (23)	
Rural	6 (8)	4 (2)	
Residents per class [mean ± SD]	7.9 ± 4.3	6.1 ± 2.9	<0.001*

a A Bonferroni correction was made such that at the α = 0.05 level, *p* values <0.0125 were considered significant and are marked with an asterisk (*).

b Urban: population (c) > 100,000; suburban: 10,000 ≤ c ≤ 100,000; rural: c < 10,000.

**Table 2:** Program Curricular and Instructional Design

Survey item	Response
Hours of didactics per week (n = 76), mean ± SD	5.2 ± 2.0
Didactic schedule (n = 76^a^), n (%)	
Daily/noon conference	48 (63)
Academic half-day	29 (37)
Asynchronous-only	0 (0)
Other	1 (1)
Resident “holds the pager” during sessions (n = 76), n (%)	
Yes	15 (20)
Somewhat	42 (55)
No	19 (25)
Who holds the pager if not junior resident (n = 61^a^), n (%)	
Senior resident	28 (46)
Attending	24 (39)
Advanced practice provider	23 (38)
Other	10 (16)
Didactic setting (n = 76), n (%)	
In-person only	6 (8)
Teleconference only	0 (0)
Hybrid/both	70 (92)
Didactics are recorded (n = 76), n (%)	52 (68)
Percent resident attendance (n = 76), mean ± SD	63.6 ± 22.0
Availability of food (n = 76), n (%)	
Always	25 (33)
More than half of the time	8 (11)
Half of the time	7 (9)
Less than half of the time	14 (18)
Rarely	22 (29)
Summary/review questions at end of session (n = 76), n (%)	
Always	6 (8)
More than half of the time	13 (17)
Less than half of the time	38 (50)
Never	19 (25)

a Multiple answers permitted per response; % is reported out of the number of responses rather than total answers selected.

### Standard Protocol Approvals, Registrations, and Participant Consents

This study protocol was reviewed and determined to be exempt from full review by the Yale Human Research Protection Program Institutional Review Board (protocol ID: 2000035052). Written informed consent to inclusion in this study was obtained from all survey respondents before initiation of the survey, and participants were given the option not to proceed or to stop the survey at any time.

### Data Availability

Full survey results will be made available by request from any qualified investigator. These data will be available for a period of at least 2 years after publication.

## Results

### Overall Program Characteristics

One-hundred eighty-one adult neurology residency training programs were identified in the AMA FREIDA database. Deliverable email addresses were found online for 178 (98%) of these program directors. Of the remaining 3 programs, 2 had at least program coordinator contact information, if not a generic program email, while the third had no valid email addresses available. Ninety-two survey responses were recorded between April 15, 2023, and July 20, 2023, representing a 50.8% initial response rate. Seventeen percent (16 of 92) of respondents did not advance beyond the initial program characteristics page of the survey and were excluded from analysis. This yielded a total of 76 survey responses included in the analysis (final response rate of 42.0%). Three of the 76 responded to only some of the survey questions (mean completion 55% for these participants), and these responses were retained for analysis.

The residency programs that participated in this survey represented a diverse group (Table 1). This convenience sample, however, was not fully representative of the general population of US adult neurology residency programs. The distribution of academic vs hybrid or community affiliation status was statistically significantly different (χ^2^ = 11.322, *p* = 0.004) with more academic programs (62 observed vs 48 expected) and fewer hybrid programs (10 observed vs 22 expected). The distribution of residency programs across regions was skewed toward the Northeast (28 observed vs 19 expected), but the difference in the overall distribution was not statistically significant (χ^2^ = 6.756, *p* = 0.147). The distribution across urban, suburban, and rural programs was statistically significantly different (χ^2^ = 13.915, *p* = 0.003) but with small effect sizes, and the largest residual was 4.5 with more rural programs observed than expected. Residency class sizes among respondents were greater than national averages (mean 7.9 vs 6.1 nationally, *p* < 0.001).

### Program Structure and Didactic Schedules

Details regarding didactic schedules are provided in Table 2. Residency programs host an average of 5.2 ± 2.0 hours of didactics weekly, with 63% of those didactics being scheduled using a daily/noon conference model while 37% of programs use an academic half-day model. Respondents reported that residents are at least “somewhat” free of the responsibility of the pager at 75% of programs, with that responsibility largely falling to some combination of the senior resident (46%), the attending neurologist (39%), or an advanced practice provider (APP) (38%). 25% of programs reported no resident pager coverage, although senior residents “held the pager” at least part time at 2 of these 19 programs. There is a teleconference option for attendance at 92% of programs, with 68% offering recordings of didactic sessions. Review or summary questions were featured at least more than half the time at 25% of programs. The mean estimated resident attendance was reported as 63.6 ± 22.0% (range 10%–90%). Food was provided by 53% of programs at least half of the time.

**Table 3:** Daily/Noon Conference vs Academic Half-Day Across Program Characteristics

Characteristic	Class	Daily/noon conference, n (%)	Academic half-day, n (%)	*p* Value
Affiliation	Academic	42 (70)	18 (30)	0.004*
	Community	2 (50)	2 (50)	0.687
	Hybrid	2 (20)	8 (80)	0.003*
Region	Northeast	24 (86)	4 (14)	0.001*
	Southeast	7 (33)	14 (67)	0.001*
	Midwest	7 (58)	5 (42)	0.767
	Southwest	5 (83)	1 (17)	0.268
	West	3 (43)	4 (57)	0.272
Setting	Urban	35 (65)	19 (35)	0.443
	Suburban	8 (57)	6 (43)	0.669
	Rural	3 (50)	3 (50)	0.524

Percentages were calculated by row. The distribution of daily/noon conference utilization vs academic half-day was compared using Mann-Whitney *U* tests. A Bonferroni correction was made such that at the α = 0.05 level, *p* values <0.0045 were considered significant and are marked with an asterisk (*).

“Other” responses to didactic scheduling included the use of both noon conference and academic half-day, daily conferences held in the morning, and 1–2-hour conferences held twice weekly. The 2 programs that mentioned they used both noon conference and academic half-day models were counted among the tallies of both. “Other” responses for who holds the pager included a fellow, a “swing” resident, and off-service rotators.

The Figure, A shows that program leaders and other faculty most frequently facilitated didactics while residents and fellows facilitated less than half of the sessions for most programs. Frequency of didactic leadership was highest for “various faculty,” followed by PDs, then residents, and fellows. The Figure, B shows that traditional lectures formed a substantial part of the didactic educational program in almost all responding programs. There was a broad range of other teaching methods used, including chalk talks, case-based discussions, and team-based learning, but each of these methods was typically used less than half the time, with case-based discussions being the second most common instructional method.

**Figure:**
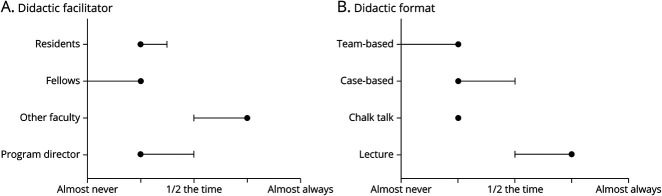
Utilization of Various Specific Didactic Facilitators and Formats (A) Plot showing the frequency with which different facilitators led resident didactic sessions. The y-axis shows responses for program directors, other faculty, fellows, and residents. The x-axis is a 5-point Likert scale ranging from almost never to almost always. The point of central tendency (black circle) for each facilitator class is the median with whiskers showing the interquartile range. (B) A similar plot showing the frequency of use for different didactic formats.

### Secondary Exploratory Analysis

Table 3 shows the distribution of daily/noon conference vs academic half-day model usage across different program affiliations, US regions, and population densities. The use of an academic half-day model was less common at academically affiliated residency programs (30.0% vs 71.4% elsewhere; *U* = 594, *p* = 0.004), more common at hybrid programs (80.0% vs 31.3% elsewhere; *U* = 164, *p* = 0.003), and similarly distributed at community-based programs. Utilization of academic half-days also varied by region, being less common in the Northeast (14% vs 52.2% elsewhere; *U* = 888, *p* = 0.001), more common in the Southeast (67% vs 26.4% elsewhere; *U* = 332, *p* = 0.001), and more evenly distributed in other geographic regions. There was no significant difference in distribution of academic half-days between urban vs suburban and/or rural setting (35.2 vs 45.0%; *H* = 0.234, *p* = 0.629). Academic half-day sessions were associated with fewer residents per class (mean 6.4 vs 8.8 with daily/noon conference; *U* = 418, *p* = 0.022). Programs following an academic half-day structure held fewer hours of didactics per week (mean 4.0 vs 5.8 with daily/noon conference; *U* = 287, *p* < 0.001).

Attendance was not significantly different with provision of food more than “rarely” (66% vs 62% elsewhere; *U* = 523, *p* = 0.415) or less than “always” (65% vs 62% elsewhere; *U* = 645, *p* = 0.934), with daily/noon conference vs academic half-day scheduling (*U* = 751, *p* = 0.230), or with the option of teleconference (*U* = 103, *p* = 0.124). Attendance was higher at programs where residents did not “hold the pager” (mean 78% vs 58% elsewhere; *U* = 262, *p* < 0.001). The number of didactic hours was inversely correlated with attendance (ρ = −0.247; *p* = 0.031). Attendance was greater where didactics were led by program directors more often (*H* = 13.358, *p* = 0.010), but not significantly different for other faculty (*H* = 3.208, *p* = 0.524), fellows (*H* = 4.586, *p* = 0.205), or residents (*H* = 8.277, *p* = 0.082). Didactic type did not significantly alter attendance, whether by lecture (*H* = 0.454, *p* = 0.929), chalk talk (*H* = 1.028, *p* = 0.794), case-based (*H* = 4.957, *p* = 0.292), or team-based (*H* = 4.821, *p* = 0.185) approaches.

The use of lectures “more than half of the time” did not vary by affiliation (*H* = 0.277, *p* = 0.871), region (*H* = 2.675, *p* = 0.614), setting (*H* = 0.603, *p* = 0.740), class size (*U* = 634, *p* = 0.768), or use of the daily/noon conference vs academic half-day model (*U* = 652, *p* = 0.915).

### Morning Report and Professor's Rounds

Table 4 provides details of morning report and professor's rounds. The programs that provided information about morning report reported a mean of 2.2 ± 1.5 conferences per week, with each lasting 46.3 ± 14.5 minutes. The most common MR presenter was the overnight resident (43%), but there was a wide range of practices. “Other” responses for MR presenter included medical students, PDs or other faculty, random residents and/or assigned residents, and off-service rotators. A similar trend was seen for the individual facilitating MR, with 43% being a PD or associate PD, but with a wide range of practices as well. A senior resident led MR at 22% of programs. Cases were “almost always” selected from within the past week at 52% of programs. Regarding learning objectives, respondents rated results interpretation and clinical reasoning of the greatest importance (4.6/5), followed by history taking and examination skills (4.1), management of the patient (4.0), and discussion of the disease after diagnosis (3.8).

**Table 4:** Morning Report (MR) and Professor's Round (PR) Practices

Survey item		Morning report	Professor's rounds
# MR per week (n = 61), mean ± SD	# PR per month (n = 37), mean ± SD	2.2 ± 1.5	2.3 ± 1.5
Session duration in min (n = 58|38), mean ± SD		46.3 ± 14.5	58.8 ± 12.6
Case presenter (n = 58|38), n (%)^a^			
Overnight resident		25 (43)	N/A
Senior resident		29 (50)	12 (32)
Junior resident		28 (48)	22 (58)
Other		16 (28)	2 (5)
Same as leader		N/A	11 (29)
Discussion leader (n = 58|38), n (%)^a^			
Senior resident		13 (22)	N/A
Consult attending		5 (9)	2 (5)
Ward attending		11 (19)	4 (11)
PD or APD		24 (41)	5 (13)
Chair or Vice Chair		8 (14)	23 (61)
Various faculty		20 (34)	16 (42)
Other		N/A	9 (24)
Case from past week (n = 58|37), n (%)			
Almost always		30 (52)	18 (49)
More than half of the time		10 (17)	8 (22)
Less than half of the time		10 (17)	8 (22)
Almost never		8 (14)	3 (8)
Learning objective importance (score out of 5), mean ± SD			
History taking/examination skills (n = 57)		4.1 ± 1.1	4.4 ± 0.8
Results interpretation and clinical reasoning (n = 58)		4.6 ± 0.7	4.5 ± 0.7
Management of the patient (n = 58)		4.0 ± 1.0	3.9 ± 1.1
Discussion of the disease after diagnosis (n = 56)		3.8 ± 1.2	4.2 ± 1.1

a Multiple answers permitted per response; % is reported out of the number of responses rather than total answers selected.

Professor's rounds were held at 38 (54%) of responding programs, at a mean frequency of 2.3 ± 1.5 sessions per month, with each lasting 58.8 ± 12.6 minutes. PR tended to be led by the department chair (39%) or various faculty (42%) and was presented by junior residents (58%) more often than senior residents (32%). At 29% of programs, the PR leader presented the case as well. Cases were “almost always” selected from within the past week at 49% of programs. Regarding learning objectives, respondents rated results interpretation and clinical reasoning of the greatest importance (4.5/5 stars), followed by history taking and examination skills (4.4), discussion of the disease after diagnosis (4.2), and management of the patient (3.9).

The total runtime of morning report per week did not vary by academic affiliation (*H* = 0.012, *p* = 0.994), region (*H* = 3.760, *p* = 0.439), setting (*H* = 2.277, *p* = 0.320), or class size (ρ = −0.043, *p* = 0.744). The incorporation of professor's rounds into didactic curricula was more common among academic residency programs (62.7% vs 8.3% elsewhere; *p* < 0.001), in the Northeast region (72.0% vs 43.5% elsewhere; *p* = 0.027), and in urban settings (67.3% vs 15.8% elsewhere; *p* < 0.001) observed using the Fisher exact test. Professor's rounds were also more common with larger residency class sizes (mean 9.4 vs 6.4 elsewhere; *U* = 936, *p* < 0.001).

### Asynchronous Learning

Table 5 provides details about asynchronous learning among respondents. Residency programs had overall positive but variable attitudes toward external asynchronous learning materials. Some encouraged (61%) or mandated (13%) the use of specific resources while 24% provided the names of resources residents may turn to for self-directed learning and 3% of respondents reported that they discouraged residents from using external resources. Most (65%) of the respondents had designed internal asynchronous learning materials, with the bulk of these focused on in-service training examination and/or board preparation (78%) and neurophysiology (i.e., EEG and EMG; 73%). 

**Table 5:** Asynchronous Learning Materials

Survey item	Response
External resources are ___ (n = 72), n (%)	
Discouraged	2 (3)
Referred to	17 (24)
Encouraged	44 (61)
Mandated	9 (13)
Program has internal resources (n = 71), n (%)	46 (65)
Internal resources by subject (n = 45), n (%)^a^	
In-service examination and/or board preparation	35 (78)
EEG and EMG	33 (73)
Entrustable professional activities	10 (22)
Career development	15 (33)
Other	11 (24)

a Multiple answers permitted per response; % is reported out of the number of responses rather than total answers selected.

“Other” responses for subjects of internal resources included billing, immunomodulatory therapy modules, wellness/humanities, movement disorders, and headache. A list of preferred external asynchronous learning materials provided by survey respondents, along with the number of mentions per resource, is included in the supplemental materials.

The use of external asynchronous resources being “encouraged” or “mandated” did not vary by academic affiliation (*p* = 0.079), region (*p* = 0.888), or setting (*p* = 0.909) using Fisher exact tests, nor by residency class size (*U* = 368, *p* = 0.081). The existence of internal resources did not vary by affiliation (*p* = 0.757), region (*p* = 0.585), or setting (0.286) using Fisher exact tests, nor by class size (*U* = 704, *p* = 0.116).

## Discussion

The results of this survey provide a cross-sectional perspective on the scope of formal educational didactic curricula across US adult neurology residency training programs. There is a broad range of educational offerings, and programs use a variety of methods to achieve their educational aims. While there are some regional and program type–specific approaches to education, the daily/noon conference approach is the most widely used overall and lectures remain a mainstay of formal residency education. Most of the programs offer hybrid in-person and remote options for participation, although this may be an immediate post–COVID-19 phenomenon.^6^

Several results from this survey provide some insight into the variety of ways that programs operationalize their educational mission. Of interest, a sizeable minority of programs reported hosting fewer than the ACGME-required 5 didactic hours per week. It is possible that differences in program director interpretation of the term “didactic” in our survey could partially explain this. For scheduling, the academic half-day model was adopted by roughly one-third of programs, more so by those with community and/or hybrid academic affiliations, those outside of the Northeast, and those with smaller numbers of residents. Many such programs were established more recently, suggesting that there may be a trend toward implementing this model in newer programs—whether from the outset or with a subsequent conversion to the model. Some older programs may be too limited by barriers including existing patient coverage delineations, availability of faculty teachers, and historical institutional culture to experiment readily with transitioning between paradigms.

It is worth noting, however, that this study was unable to evaluate whether academic half-days vs daily/noon conferences led to different educational outcomes for residents or different clinical outcomes for patients. Previous studies of internal medicine and pediatrics programs have demonstrated that a transition from a daily/noon conference format to an academic half-day may result in improved attendance, protected time for learning, resident wellness, and overall satisfaction in their educational experience.^7-9^ One experimental study from Internal Medicine even concluded that residents with an academic half-day curriculum had greater knowledge attainment when measured using in-service training examination scores.^10^ Academic half-days were not associated with increased attendance in our study—although these other outcomes above including examination scores were not collected for analysis. Our study also did not support that academic half-day usage led to increased utilization of nonlecture didactic instructional design. The use of “flipped classroom” methods also did not seem to affect reported resident attendance, but this could be partially due to low rates of use of these methods overall. Therefore, in our study, it is not clear that either academic half-day or daily/noon conference scheduling format necessarily drove resident attendance or educational innovation on the part of program leadership.

Notably, factors that did significantly increase resident attendance were residents not “holding the pager,” fewer total hours of conferences, and program directors frequently leading teaching sessions. It is conceivable that other “core” teaching faculty members may similarly draw greater resident attendance, although this was not specifically measured. Three-quarters of programs responded that residents were at least “somewhat” protected from holding the pager, with that responsibility being assumed slightly more often by senior residents followed by attendings and APPs. At one-quarter of programs, there seems to have been a more concerted effort to liberate even senior residents of their pager-bearing responsibilities. This educational environment may have promoted not only attendance but also resident engagement and knowledge formation by reducing “extraneous cognitive load” from distractions and permitting greater attentional energy to be applied to the learning material.^11^ A challenge of this paradigm, however, is that covering attending physicians have previously raised concerns that increased transitions of care surrounding academic half-days may disrupt residents' situated learning, break patient continuity of care, and potentially affect patient workflow and safety.^7^ This may also exacerbate strains on teaching faculty time and burnout. Ultimately, programs will have to decide at their own institutions whether dedicating the time, personnel, and ultimately financial resources to protecting educational resident time is feasible within their setting and a justifiable use of limited resources. Another financial consideration concerns the provision of conference food, which may constitute a significant expenditure at some institutions. Our study did not support that providing food more often led to increased resident didactic attendance. Another study that directly tracked individual resident attendance, however, rather than relying on PD report as in this study, found that lunch delivery did significantly improve conference attendance and punctuality.^12^

The diversity of practices and primary discussants surrounding case-based conferences such as morning reports and professor's rounds was striking. It was particularly interesting to see the different weighing of learning objective priorities between these 2 conference types, with discussion of the underlying disease overtaking discussion of patient management with professor's rounds.

Most participating residency programs reported having developed internal asynchronous learning materials. It is perhaps unsurprising that most of these relate to in-service and/or board examination preparation and neurophysiology techniques. In-service and board examinations are high-stakes assessments and thus associated with a high motivation to engage with asynchronous and other educational materials. Direct clinical neurophysiology exposure is often difficult to incorporate into inpatient rotations, and there are several reports of insufficient EEG and EMG training in residency, thus making asynchronous materials essential in many cases.^13,14^ Beyond filling an educational gap, one study of medical students found that online modules may promote greater electrodiagnostic interpretation skills than standard lectures when assessing EKGs.^15^ Development of asynchronous learning materials requires a high amount of initial investment regarding time and cognitive energy; there is likely an opportunity for more efficient sharing of these resources between programs, although communication and technological barriers exist. Shared asynchronous (and synchronous) resources could reduce this burden and allow for more faculty and resident time spent interacting and sharing their experiences and skills.^16^ Overall, while three-quarters of programs encouraged the use of external resources, it was interesting that the remaining programs were passively supportive of their use while a minority of programs discouraged their use. Resident attitudes toward online asynchronous learning from elsewhere in the literature, however, tend to be very positive with high levels of regular engagement.^17-19^

Based on our findings, we would recommend the following for program directors and other stakeholders designing formal didactic curricula for adult neurology residency training programs:

1. Consider relieving residents of pager responsibilities as much as is safe for patient care and feasible within the department considering available resources. This tends to improve attendance and may increase engagement by reducing cognitive load.
2. Consider avoiding overscheduling teaching sessions because this negatively affects attendance.
3. Consider including more didactics in the curriculum led by program directors or other core teaching faculty because this tends to improve attendance.
4. Programs are encouraged to experiment with alternative teaching methods such as “flipped classrooms” and other innovative strategies because there was limited use of non lecture-based formats found in this study.
5. In addition, residency programs may review the list of online asynchronous resources provided as a supplement to this article. These were identified by program directors as quality materials to support residents' independent study.

This study has several limitations. While the relatively high response rate for this type of national survey strengthens the internal validity of this study, there was an over-representation of large academic centers. Readers from such institutions, therefore, can be reassured that these results hold true for their nearest “peer” institutions, but the same cannot be said for all programs. This also raises the possibility that potentially contrasting curricular practices at less well-represented programs may have not been captured. It is also worth noting that residency programs housed at large academic centers may tend to be better resourced than the national cohort of residency programs, so curricular practices at these centers may be reflective of this reality as much as of local culture. Another limitation of this survey is that duplicate answers may have been recorded because individual responses were not tracked. This is not likely to have a large effect because it is believed that most program directors would have remembered previously responding and, therefore, avoided completing the questionnaire a second time. This survey intentionally did not mark any questions as “mandatory” to limit the attrition of programs whose directors felt unable or unwilling to answer a particular question. There is the possibility that this method decreased our ability to capture a true cross-section of responses for all items. It is also worth noting that actual resident attendance was not obtained by this survey and program directors' report of overall percentage attendance may be inaccurate for any of several reasons.

Finally, this study is descriptive only and is, therefore, not constructed to identify or comment on best practices. No clinical or educational outcomes were measured, so we are not able to correlate specific programmatic practices with resident clinical or academic performance—including on examinations. Further studies would be invaluable to evaluate best practices and explore the perspectives of all relevant stakeholders. There is a broad range of work investigating many aspects of formal classroom-based instruction in graduate medical education, and there is room for ongoing exploration into the processes behind and purposes of such instruction.^20^ The purpose of didactics extends far beyond simple knowledge acquisition and includes providing a space for educational growth and the formation of a learning community that is not primarily driven by patient care or other systemic needs.^20^ Future work in neurology education could delve further into the effectiveness of didactic instructional techniques from these perspectives, the methods that foster the growth of learning communities, and an understanding of how didactics fit into the overall educational culture of neurology residency. While our study provides a useful perspective on the overall structures of didactic education in US neurology residency programs, there is clearly an opportunity for further discussions about optimal practices and how to share resources and experiences so that every program can build on its strengths and adapt to its individual circumstances.

## Glossary

ACGME: Accreditation Council for Graduate Medical Education
AHD: academic half-day
GME: graduate medical education
NC: noon conference
PD: program director

## References

[1] Kolb D. Experiential Learning: Experience as the Source of Learning and Development. Journal of Business Ethics; 1984.

[2] Jean Lave EW. Situated Learning: Legitimate Peripheral Participation. Cambridge University Press; 1991.

[3] ACGME Program Requirements for Graduate Medical Education in Neurology. Accreditation Council for Graduate Medical Education (ACGME); 2023. Accessed February 15, 2025. https://www.acgme.org/specialties/neurology/program-requirements-and-faqs-and-applications/.

[4] Zimmerman WD, Pergakis MB, Gorman EF, et al. Scoping review: innovations in clinical neurology education. Neurol Educ. 2023;2(1):e200048. doi:10.1212/NE9.0000000000200048 39411110 PMC11473089

[5] Artino AR Jr., La Rochelle JS, Dezee KJ, Gehlbach H. Developing questionnaires for educational research: AMEE Guide No. 87. Med Teach. 2014;36(6):463-474. doi:10.3109/0142159X.2014.889814 24661014 PMC4059192

[6] Hahn TW. Virtual noon conferences: providing resident education and wellness during the COVID-19 pandemic. PRiMER. 2020;4:17. doi:10.22454/PRiMER.2020.364166 33111044 PMC7581201

[7] Batalden MK, Warm EJ, Logio LS. Beyond a curricular design of convenience: replacing the noon conference with an academic half day in three internal medicine residency programs. Acad Med. 2013;88(5):644-651. doi:10.1097/ACM.0b013e31828b09f4 23524926

[8] Zastoupil L, McIntosh A, Sopfe J, et al. Positive impact of transition from noon conference to academic half day in a pediatric residency program. Acad Pediatr. 2017;17(4):436-442. doi:10.1016/j.acap.2017.01.009 28130128

[9] Randall MH, Schreiner AD, Clyburn EB, Rockey DC, Duckett A. Effects of an academic half day in a residency program on perceived educational value, resident satisfaction and wellness. Am J Med Sci. 2020;360(4):342-347. doi:10.1016/j.amjms.2020.05.013 32553748

[10] Ha D, Faulx M, Isada C, et al. Transitioning from a noon conference to an academic half-day curriculum model: effect on medical knowledge acquisition and learning satisfaction. J Grad Med Educ. 2014;6(1):93-99. doi:10.4300/JGME-D-13-00185.1 24701317 PMC3963802

[11] Van Merriënboer JJG, Sweller J. Cognitive load theory in health professional education: design principles and strategies. Med Educ. 2010;44(1):85-93. doi:10.1111/j.1365-2923.2009.03498.x 20078759

[12] Cosimini MJ, Mackintosh L, Chang TP. Number needed to eat: pizza and resident conference attendance. Med Educ. 2016;50(12):1204-1207. doi:10.1111/medu.13080 27873408

[13] Nascimento FA, Gavvala JR. Education research: neurology resident EEG education: a survey of US Neurology Residency Program Directors. Neurology. 2021;96(17):821-824. doi:10.1212/WNL.0000000000011354 33310878

[14] Daniello KM, Weber DJ. Education research: the current state of neurophysiology education in selected neurology residency programs. Neurology. 2018;90(15):708-711. doi:10.1212/WNL.0000000000005296 29632112

[15] Olvet DM, Sadigh K. Comparing the effectiveness of asynchronous e-modules and didactic lectures to teach electrocardiogram interpretation to first year US medical students. BMC Med Educ. 2023;23(1):360. doi:10.1186/s12909-023-04338-6 37217893 PMC10201768

[16] Le TT, Prober CG. A proposal for a shared medical school curricular ecosystem. Acad Med. 2018;93(8):1125-1128. doi:10.1097/ACM.0000000000002194 29517524

[17] Mallin M, Schlein S, Doctor S, Stroud S, Dawson M, Fix M. A survey of the current utilization of asynchronous education among emergency medicine residents in the United States. Acad Med. 2014;89(4):598-601. doi:10.1097/ACM.0000000000000170 24556776 PMC4885578

[18] Wittich CM, Agrawal A, Cook DA, et al. E-learning in graduate medical education: survey of residency program directors. BMC Med Educ. 2017;17(1):114. doi:10.1186/s12909-017-0953-9 28697744 PMC5504987

[19] Kelleher M, Miller RE, Duckett A, et al. Self-directed learning among internal medicine residents in the information age. South Med J. 2020;113(9):457-461. doi:10.14423/SMJ.0000000000001144 32885266

[20] Chen LYC, Quach TTT, Dayan R, Giustini D, Teunissen PW. Academic half days, noon conferences and classroom-based education in postgraduate medical education: a scoping review. CMAJ Open. 2023;11(3):E411-e425. doi:10.9778/cmajo.20210203 PMC10174266 37160324

